# Pathways to Homelessness among Older Homeless Adults: Results from the HOPE HOME Study

**DOI:** 10.1371/journal.pone.0155065

**Published:** 2016-05-10

**Authors:** Rebecca T. Brown, Leah Goodman, David Guzman, Lina Tieu, Claudia Ponath, Margot B. Kushel

**Affiliations:** 1 Division of Geriatrics, University of California, San Francisco, San Francisco, California, United States of America; 2 San Francisco Veterans Affairs Medical Center, San Francisco, California, United States of America; 3 School of Medicine, University of California, San Francisco, San Francisco, California, United States of America; 4 Division of General Internal Medicine, University of California, San Francisco, Zuckerberg San Francisco General Hospital and Trauma Center, San Francisco, California, United States of America; 5 Center for Vulnerable Populations at Zuckerberg San Francisco General Hospital and Trauma Center, University of California, San Francisco, San Francisco, California, United States of America; Cardiff University, UNITED KINGDOM

## Abstract

Little is known about pathways to homelessness among older adults. We identified life course experiences associated with earlier versus later onset of homelessness in older homeless adults and examined current health and functional status by age at first homelessness. We interviewed 350 homeless adults, aged 50 and older, recruited via population-based sampling. Participants reported age at first episode of adult homelessness and their life experiences during 3 time periods: childhood (<18 years), young adulthood (ages 18–25), and middle adulthood (ages 26–49). We used a structured modeling approach to identify experiences associated with first adult homelessness before age 50 versus at age 50 or older. Participants reported current health and functional status, including recent mental health and substance use problems. Older homeless adults who first became homeless before 50 had more adverse life experiences (i.e., mental health and substance use problems, imprisonment) and lower attainment of adult milestones (i.e., marriage, full-time employment) compared to individuals with later onset. After multivariable adjustment, adverse experiences were independently associated with experiencing a first episode of homelessness before age 50. Individuals who first became homeless before age 50 had higher prevalence of recent mental health and substance use problems and more difficulty performing instrumental activities of daily living. Life course experiences and current vulnerabilities of older homeless adults with first homelessness before age 50 differed from those with later onset of homelessness. Prevention and service interventions should be adapted to meet different needs.

## Introduction

Over the past 25 years, the average age of single adults experiencing homelessness has increased [1]. Today, approximately half of single homeless adults are aged 50 and older [2], compared to 11% in 1990 [1]. Recent evidence suggests that this trend is due to a cohort effect: individuals born in the second half of the baby boom (1954–1963) have had an elevated risk of homelessness throughout their lives, and are now aged 50 and older [2]. Homeless adults aged 50 and older have unique health problems compared to younger homeless adults, including memory loss, functional impairment, and falls, conditions more commonly seen in housed adults aged 70 and older [3–6]. Due to the premature development of multiple health concerns in homeless adults, researchers have considered homeless adults to be “older” at age 50 [3,7,8]. There has been a growing call to develop strategies to prevent and end homelessness among older adults [9,10], particularly as increasing numbers of older adults face severe housing cost burden, defined as spending 50% or more of household income on housing costs [11].

Research on older homeless adults conducted in England twenty years ago suggested that there are different risk factors for and triggers of homelessness for individuals who entered homelessness in early adulthood, middle age, and late life. Those who became homeless in early life had experienced breakdown of the family home; those who became homeless in mid-life had experienced the death of a parent, breakdown of a relationship, or loss of work; and those who became homeless in late life had experienced the death of a spouse, retirement, the loss of housing tied to employment, or worsened mental health problems or cognitive impairments. [7,12] However, little is known about pathways to homelessness among older homeless adults in the United States since the change in demographics of the homeless population.

When the homeless population was younger, homelessness was thought to stem from a gradual accumulation of personal problems beginning in childhood—e.g., out-of-home experiences, low educational and occupational attainment, mental health and substance use problems—leading to homelessness in early adulthood [7,13]. However, recent studies suggest that up to half of older homeless adults become homeless for the first time in late middle age. These newly homeless individuals are generally low-income adults who sustained a financial or health crisis after a lifetime of workforce participation and housing [14,15]. These problems may be accentuated by a shortage of subsidized housing for older adults living in poverty, a lack of employment options for semi-skilled and unskilled laborers in late middle-age, and the inability to collect income entitlements before age 65 [7,16,17]. These findings suggest that older homeless adults represent a diverse group with distinct life course experiences. However, little is known about pathways to homelessness among older homeless adults with early versus late life entry into homelessness.

There is growing appreciation of the need to understand health through a life course approach which recognizes that there are specific critical periods of growth and development. Conceptual models of the life course include those that examine critical periods and others that examine accumulated risk; there is evidence for each [18]. Models that take into account critical periods assume that an insult during a specific period has lifetime effects, which may be modified by later life factors. Models that rely on risk accumulation assume that risk factors at each stage combine to create risk. Little is known about whether negative life events act according to a critical period model or a risk accumulation model to lead to homelessness at different life stages [19–21].

Individuals with differing life experiences may have different needs and strengths, and thus need different services and interventions. Therefore, we assessed the life course experiences and the current health and functional status of 350 homeless adults aged 50 and over, and the age when these individuals first experienced adult homelessness. We hypothesized that experiencing negative life events (e.g., substance use problems, imprisonment) and having lower attainment of typical milestones in young and middle adulthood (e.g., marriage, steady employment) would be more common in individuals with onset of first homelessness before age 50. In exploratory analyses, we examined whether these life experiences were associated with first homelessness via a critical period model or a risk accumulation model. We further examined the prevalence of current vulnerabilities in the cohort, including mental health and substance use problems, and hypothesized that individuals with earlier onset of homelessness would have higher rates of current vulnerabilities compared to those with later onset of homelessness.

## Methods

### Design overview

We interviewed 350 homeless adults aged 50 and older recruited via population-based sampling in Oakland, California. These were enrollment interviews for a prospective cohort study, Health Outcomes in People Experiencing Homelessness in Older Middle agE (HOPE HOME). We developed the study methods in partnership with a community advisory board. The institutional review board of the University of California, San Francisco, approved the study.

### Sample

We sampled homeless individuals using a purposive population-based sampling method similar to that of our previous research [22]. We extended our prior sampling frame to include recycling centers and homeless encampments in order to represent unsheltered individuals adequately. The sampling locations included all low-cost meal programs serving homeless people at least 3 meals weekly (n = 5), all overnight shelters serving single homeless adults aged 25 and older (n = 5), a recycling center, and places where unsheltered people stayed overnight. Study staff used standard protocols to recruit individuals randomly from each site, and set sampling goals for each site based on best estimates of the number of individuals who visited a site or were unsheltered annually. Individuals who met a brief eligibility screen were invited to participate in an enrollment interview.

### Enrollment and baseline interviews

Study staff conducted enrollment and baseline interviews between July 2013 and June 2014 at a non-profit community center in Oakland. Individuals were eligible if they were aged 50 or older, English speaking, and currently homeless based on the federal Homeless Emergency Assistance and Rapid Transition to Housing (HEARTH) Act [23].

After assessing eligibility, staff obtained written informed consent using a teach-back method [24]; individuals unable to provide consent were excluded. This consent procedure was approved by the institutional review board of the University of California, San Francisco. Eligible individuals completed in-depth structured interviews. Individuals received a $25 gift card for completing eligibility and baseline interviews.

**Fig 1:**
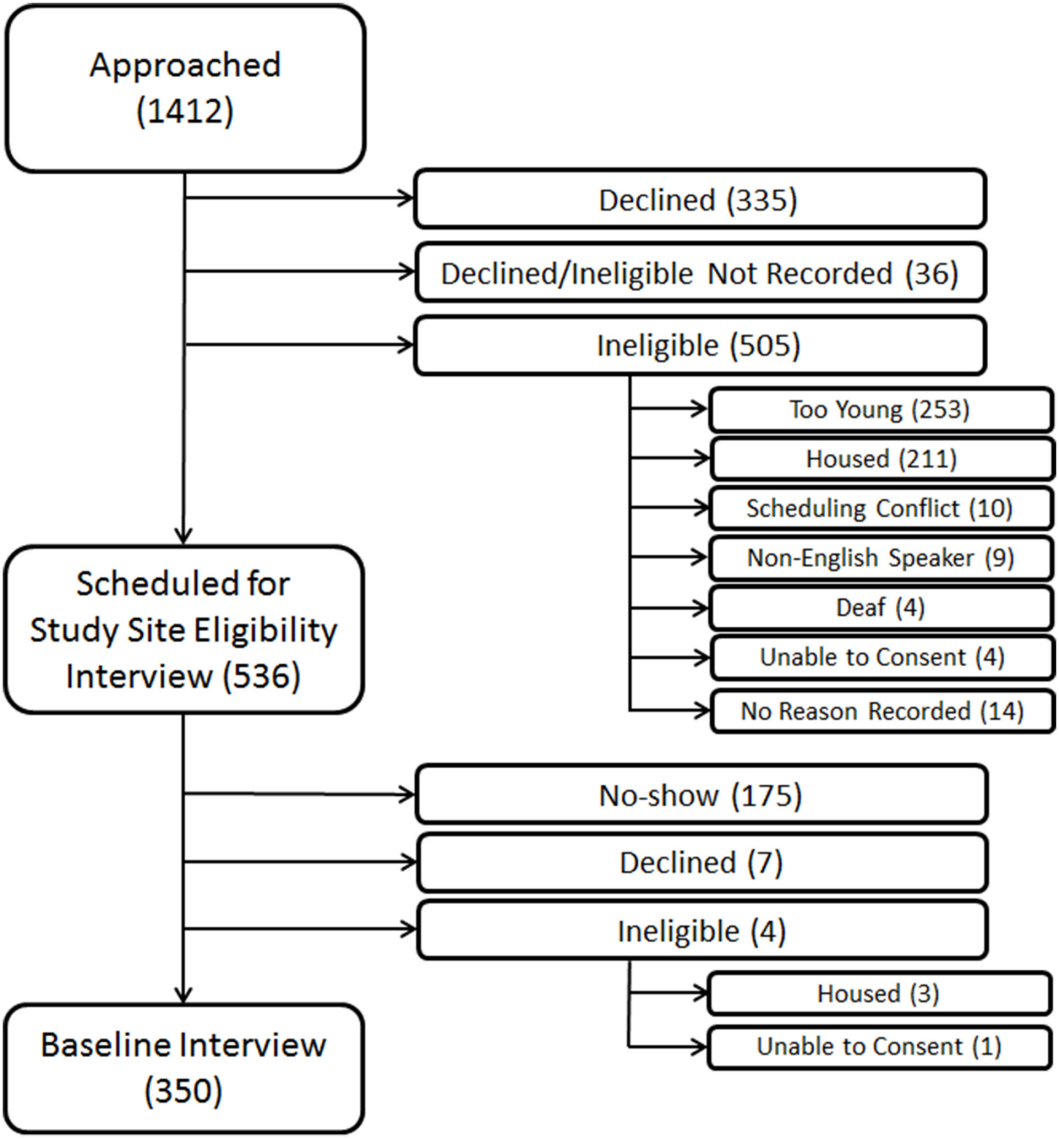
Flow-chart of recruitment of 350 older homeless adults. This figure shows the number of individuals approached, assessed for eligibility, and enrolled in the study (N = 350), noting specific reasons for inability to enroll. Values represent the number of individuals in each group. *Participants who declined after being approached (N = 335) declined before being assessed for eligibility. Therefore, the number of participants who were ineligible for the study may have been higher than the numbers presented in this figure.

Individuals who declined participation or did not attend the interview were similar to enrolled participants by sex, but were more likely to be African-American by observed race/ethnicity (82.3% vs. 79.7%, P = .04) and more likely to be recruited from meal programs (55.3% vs. 49.1%) and from unsheltered areas or recycling centers (20.1% vs. 15.7%, overall P value for comparison = 0.003) (Fig 1).

### Measures

#### Age at first adult homelessness

Our dependent variable was the age at which an individual first experienced adult homelessness, determined by self-report. We dichotomized reported ages as younger than 50 years versus 50 years or older.

#### Sociodemographic characteristics

Participants reported their age, gender, race/ethnicity (African-American, white, Latino, other) and highest level of education. We defined low educational attainment as lacking either a high school or General Educational Development (GED) diploma. Participants reported their usual occupation; we defined low occupational status as usually working as an unskilled laborer, categorized using the Hollingshead Categories [25] as adapted by the Addiction Severity Index [26]. The Addiction Severity Index included seven occupational categories: higher executives, business managers, administrative personnel and small business owners, clerical and sales workers, skilled manual workers, semi-skilled workers, and unskilled laborers [26]. Participants reported their total lifetime years of homelessness and the number of months of their current episode of homelessness.

#### Life course measures

Participants reported life course experiences during 3 time periods: childhood (age <18), young adulthood (ages 18–25), and middle adulthood (ages 26–49).

Adverse childhood experiences: Participants reported if a parent or caregiver died during their childhood or if a parent was incarcerated for 12 months or longer. We adapted questions from the National Survey of Homeless Assistance Providers and Clients (NSHAPC) to ask participants if they experienced any of the following out-of-home experiences before the age of 18: (1) incarceration in the juvenile justice system, (2) placement in the child welfare system (combining the separate age categories in NSHAPC to one timeframe of ages 0–18), and (3) homelessness after running away or being forced to leave home for over 24 hours (combining the 2 separate age categories in NSHAPC to one category and asking a follow-up question to assess homelessness following this episode) [27]. We asked participants if they ever experienced homelessness as a child with their families. Participants reported if they had experienced neglect, emotional abuse, physical abuse, or sexual abuse before age 18 [28].

Sociodemographic and health-related life course experiences: Other life course measures included socioeconomic attainment, marriages/partnerships, imprisonment, health status, and health-related behaviors. For each measure, we specify during which life course period it was collected.

We assessed socioeconomic attainment during young and middle adulthood. Participants reported if they either had difficulty paying bills or received government support during each life course period. Participants reported the number of years that they were employed (working at least 20 hours a week) during each life course period. We defined participants as underemployed during the period if they were employed for less than half the time period. Participants reported any marriages or life-partnerships during young and middle adulthood. Participants also reported if, and during which years, they had been imprisoned in a state or federal prison as an adult. We categorized each of these measures as any versus none for each time period.

Participants reported if a health care provider had ever told them that they had any of the following chronic medical conditions, and, if so, their age at diagnosis: hypertension, coronary artery disease, myocardial infarction, or angina, congestive heart failure, stroke or transient ischemic attack, diabetes, asthma or chronic obstructive pulmonary disease, HIV or AIDS, or cancer [29]. We defined participants as having a chronic medical condition during a given life course period (childhood, young adulthood, and middle adulthood) if they were diagnosed with one of these conditions during that life course period.

Participants reported any episodes of traumatic brain injury, defined as having hit or been hit on their head with associated loss of consciousness, and the age when they experienced each injury [30]. We defined participants as having a traumatic brain injury during each life course period when they reported an injury.

To assess history of mental health problems, we adapted the lifetime mental health measures from NSHAPC [27] and the Addiction Severity Index [26] to ask at what age any problems first occurred. Participants reported if they had ever experienced serious anxiety, depression, difficulty controlling violent behavior, or hallucinations unrelated to substance use; had attempted suicide; or had been prescribed medication by a doctor for psychiatric problems, and if so, at what age [27]. We considered participants to have a mental health problem during the life course period when they first reported the problem.

To assess problematic use of alcohol and other substances, we adapted the lifetime substance use questions from the Addiction Severity Index [26] to ask participants to report number of lifetime years of regular alcohol and drug use. We also asked participants to report at what age they first began to use a substance regularly. For alcohol we asked participants to report use during 4 age ranges: before age 18, 18–25 years, 26–49 years, and 50 years and above. For all other substances, we calculated whether the participant was using a substance regularly during a particular age period by adding the years of regular use to the age when he or she started to use regularly. We defined a history of alcohol use problems as reporting regular drinking (drinking to get drunk 3 or more times a week), and a history of drug use problems as reporting regular drug use (using drugs 3 or more times a week) [26].

#### Current vulnerability measures

To assess differences in current vulnerabilities according to onset of homelessness, we examined measures of incarceration, current health status, mental health status, substance use, functional status, and cognitive status.

Incarceration: We asked participants if they had ever spent time in jail or prison and if so, when this most recently occurred, and created a binary variable for incarceration within the past 6 months.

Health status: To assess current health status, participants rated their general health (fair or poor versus good, very good, or excellent) [31] and reported if a health care provider had ever told them they had hypertension, coronary artery disease, myocardial infarction, or angina, congestive heart failure, stroke or transient ischemic attack, diabetes, asthma or chronic obstructive pulmonary disease, HIV or AIDS, or cancer [32].

Mental health status and substance use: We assessed depressive symptoms using the Center for Epidemiologic Studies Depression Scale, categorizing scores of ≥22 as indicative of moderate to severe depressive symptoms [33]. We screened participants for post-traumatic stress disorder (PTSD) using the Primary Care PTSD Screen, categorizing scores of 3 or more as a positive screen for PTSD [34]. We used questions from the National Survey of Homeless Assistance Providers and Clients to determine if participants had experienced any of the following problems in the prior 6 months: (1) hallucinations; (2) trouble controlling violent behavior; or (3) attempted suicide [27]. Participants reported if they had received outpatient mental health treatment in the past 6 months or had been hospitalized for a mental health problem in the past 6 months.

To assess risk and severity of current alcohol use disorders, we used the WHO's Alcohol Use Disorders Identification Test (AUDIT), with a shortened timeframe of the previous 6 months rather than the previous 12 months [35]. To assess drug use problems, we used questions from the World Health Organization’s (WHO) Alcohol, Smoking, and Substance Involvement Screening Test (ASSIST), using a lengthened timeframe of the previous 6 months rather than the previous 3 months [36]. We defined participants with moderate or greater severity illicit drug use (ASSIST score ≥4) as having at-risk drug use [35], and hazardous and harmful alcohol use or greater (AUDIT score ≥8) as having at-risk alcohol use [36].

Functional and cognitive status: Participants reported if they had difficulty performing 5 activities of daily living (ADLs; bathing, dressing, eating, transferring, toileting) [37], and 6 instrumental activities of daily living (IADLs; taking transportation, managing medications, managing money, applying for benefits, setting up a job interview, finding a lawyer) [38]. We defined ADL impairment as difficulty performing 1 or more ADLs; we defined IADL impairment similarly. We defined mobility impairment as self-reported difficulty walking across a room [37].

We assessed cognitive impairment using the Modified Mini-Mental State Examination [32]. We defined cognitive impairment as a score below the 7^th^ percentile (i.e., 1.5 standard deviations below a reference cohort mean) or inability to complete the assessment [32,39].

#### Statistical analyses

We examined participant characteristics and life course experiences using descriptive statistics. We then identified life course experiences associated with experiencing a first episode of adult homelessness before age 50 versus at age 50 or older. We evaluated four models representing alternative hypothetical associations between each life course experience and experiencing a first episode of adult homelessness before age 50: 1) three models, each with a single dichotomous variable representing exposure during one life course period (described by Mishra as the critical period model) [40]; (2) a model with dichotomous terms for all three periods; (3) a model with the three dichotomous terms plus interaction terms for each pair of life course variables; and (4) a model with a cumulative score variable representing the number of life course periods the exposure was experienced (range, 0–3; described by Mishra as the accumulation model) [40].

Some of these variables, specifically the cumulative scores, are linear combinations of the critical period dichotomous variables. For this reason, it is not possible to begin with a multivariate model containing terms for all four of the hypothetical associations described above and then use backward elimination or another typical model building strategy to compare the various hypotheses. Therefore, we used a strategy roughly following that of Mishra to select the best fitting model for each variable [40]. We used log likelihood statistics and predicted versus observed probabilities of the outcome to compare the alternative models with a saturated model containing all the terms needed to define the four hypothetical associations described above. In cases where two models were similar in terms of fit, we further assessed model fit using the Akaike Information Criterion (AIC) [41]. After selecting the best bivariate model for each exposure, we entered those life course variables into a multivariable model along with covariates with bivariate P values of 0.20 or less. We reduced the multivariable model using backward elimination until only variables with P values of 0.05 or less remained.

## Results

### Participant characteristics and life course experiences overall and dichotomized by age at first homelessness

The median age of participants was 58 years (range, 50–80 years; IQR, 54, 61), 77.1% were male, 79.7% were African American, and 25.7% had not completed high school or obtained a GED. More than forty percent of participants (43.4%) experienced their first episode of adult homelessness at age 50 or older.

Participant characteristics and life course experiences differed by age at first adult homelessness. Compared to individuals whose first homelessness occurred at age 50 or older, individuals with first homelessness before age 50 had lower educational attainment (31.3% less than high school education/GED versus 18.4%, P = .006) and longer duration of lifetime homelessness (4.2 years [IQR, 1.7, 10.0] versus 2.0 years [IQR, 0.7, 5.3]) (Table 1). Of individuals with first homelessness before age 50, 73.0% had been continuously homeless for 1 year or longer in their current episode of homelessness, compared to 59.9% of those with first homelessness at age 50 or older (P = .01). During childhood, individuals whose first episode of adult homelessness occurred before age 50 had a higher prevalence of incarceration in the juvenile justice system compared to those with first homelessness after age 50 (27.8% versus 15.8%, P = .008), as well as a higher prevalence of childhood abuse or neglect (66.8% versus 54.6%, p = .02), chronic illness diagnosed in childhood (13.1% versus 3.3%, P = .001), and drug use problems in childhood (48.0% versus 31.6%, P = .002) (Table 1).

**Table 1:** Baseline characteristics and life course experiences of 350 older homeless adults by age at first homelessness.

Characteristic	First homelessness before age 50 (n = 198)	First homelessness age 50 or older (n = 152)	P value
**Sociodemographics**			
Male, no. (%)	154 (77.8)	116 (76.3)	0.75
Race/ethnicity, no. (%)			0.76
African American	157 (79.3)	122 (80.3)	
White	24 (12.1)	14 (9.2)	
Latino	7 (3.5)	9 (5.9)	
Other	8 (4.0)	6 (3.9)	
Less than high school education or GED, no. (%)	62 (31.3)	28 (18.4)	0.006
Usual occupation as unskilled laborer, no. (%)	63 (32.6)	38 (25.0)	0.12
Years of lifetime homelessness, median (IQR)	4.2 (1.7, 10.0)	2.0 (0.7, 5.3)	<.001
Homeless for 1 year or longer during current episode of homelessness, no. (%)	143 (73.0)	91 (59.9)	0.01
**Childhood adverse experiences (<18 years), no. (%)**			
Single parent household	101 (51.5)	68 (45.0)	0.23
Death or incarceration of caregiver	48 (24.2)	38 (25.0)	0.90
Death of caregiver	41 (20.8)	36 (23.8)	0.50
Caregiver incarcerated or imprisoned for at least 12 months	14 (7.2)	5 (3.3)	0.12
Out-of-home experience			
Placement in child welfare system	21 (10.6)	8 (5.3)	0.07
Incarceration in juvenile justice system	55 (27.8)	24 (15.8)	0.008
Homelessness experienced with one’s family	7 (3.5)	1 (0.7)	0.07
Homelessness after running away/being forced to leave home	32 (52.5)	9 (31.0)	0.06
Abuse or neglect^a^	131 (66.8)	83 (54.6)	0.02
Verbal abuse	109 (55.3)	63 (41.4)	0.01
Physical abuse	72 (36.5)	44 (29.1)	0.15
Sexual abuse	34 (17.3)	12 (7.9)	0.01
Neglect	31 (15.8)	7 (4.6)	<.001
Any chronic medical condition^b^	26 (13.1)	5 (3.3)	0.001
Traumatic brain injury	24 (12.1)	21 (13.8)	0.64
Mental health problem^d^	154 (77.8)	117 (77.0)	0.86
Alcohol use problem^e^	113 (58.2)	81 (54.4)	0.47
Drug use problem^f^	95 (48.0)	48 (31.6)	0.002
**Young adulthood (18–25 years), no. (%)**			
Difficulty paying bills or received government support	66 (33.3)	24 (15.8)	<.001
Underemployed^c^	77 (40.5)	46 (32.2)	0.12
Lacked spouse/partner	86 (45.7)	52 (35.6)	0.06
Imprisoned in a state or federal prison	116 (58.6)	66 (43.4)	0.005
Any chronic medical condition^b^	16 (8.1)	6 (3.9)	0.11
Traumatic brain injury	14 (7.1)	9 (5.9)	0.67
Mental health problem^d^	50 (25.3)	23 (15.1)	0.02
Alcohol use problem^e^	92 (47.7)	45 (30.4)	0.001
Drug use problem^f^	138 (69.7)	82 (53.9)	0.003
**Middle adulthood (26–49 years), no. (%)**			
Difficulty paying bills or received government support	131 (66.2)	78 (51.3)	0.005
Underemployed^c^	101 (54.3)	42 (29.0)	<.001
Lacked spouse/partner	42 (22.3)	22 (15.1)	0.09
Imprisoned in a state or federal prison	118 (59.6)	75 (49.3)	0.06
Any chronic medical condition^b^	76 (38.4)	45 (29.6)	0.09
Traumatic brain injury	44 (22.2)	14 (9.2)	0.001
Mental health problem^d^	71 (35.9)	44 (28.9)	0.17
Alcohol use problem^e^	105 (54.7)	69 (47.3)	0.18
Drug use problem^f^	149 (75.3)	90 (59.2)	0.001

^a^Abuse or neglect defined as experiencing either neglect, emotional abuse, physical abuse, or sexual abuse before the age of 18.

^b^Chronic medical condition defined as receiving a new diagnosis of any of the following during a given life course period: coronary artery disease or myocardial infarction; congestive heart failure; stroke or transient ischemic attack; diabetes; asthma or chronic obstructive pulmonary disease; arthritis; HIV or AIDS, or cancer.

^c^Underemployment during a given life course period defined as working at least 20 hours per week less than half the time during that time period.

^d^Mental health problem defined as first reporting any of the following experiences during a given life course period: experiencing serious anxiety, depression, difficulty controlling violent behavior, or hallucinations that were not the result of substance use; attempting suicide; or being prescribed medication by a doctor for psychiatric problems.

^e^Alcohol use problem defined as a self-reported history of drinking to get drunk 3 or more times a week during a given life course period.

^f^Drug use problem defined as self-reported use of drugs 3 or more times a week during a given life course period.

Life course experiences also differed in young adulthood (18–25 years). Individuals whose first homelessness occurred before age 50 reported a higher prevalence of imprisonment during young adulthood (58.6% versus 43.4%, P = .005), as well as a higher prevalence of mental health problems (25.3% versus 15.1%, P = .02), alcohol use problems (47.7% versus 30.4%, P = .001), and drug use problems (69.7% versus 53.9%, P = .003).

In middle adulthood (26–49 years), individuals with first homelessness before age 50 had a higher prevalence of underemployment (54.3% versus 29.0%, P <.001), traumatic brain injury (22.2% versus 9.2%, P = .001), and drug use problems (75.3% versus 59.2%, P = .001).

#### Life course experiences associated with first homelessness before age 50 versus at age 50 or older

Model selection: For five life experiences, a model including a single dichotomous variable in young or middle adulthood provided the best fit, including receiving government support or having difficulty paying bills; lacking a spouse/partner; having mental health problems; being underemployed; and having a traumatic brain injury (Table 2). For four other life course experiences (being imprisoned; being diagnosed with a new chronic medical condition; experiencing alcohol use problems; experiencing drug use problems), a model with a cumulative score variable provided the best model fit, in which the score represented the total number of life course periods (childhood, young adulthood, middle adulthood; range, 0–3) in which a participant experienced the exposure.

**Table 2:** Life course experiences associated with first episode of homelessness before age 50 versus at age 50 or older among 350 older homeless in Oakland, California.

Characteristic	Bivariable odds ratio for first homelessness before age 50 versus at age 50 or older (95% CI)	Multivariable odds ratio for first homelessness before age 50 versus at age 50 or older^a^ (95% CI)
**Sociodemographic characteristics**		
Woman	0.92 (0.55–1.5)	
African American	0.94 (0.55–1.6)	
Less than high school education or GED	2.0 (1.2–3.4)	
Usual occupation as unskilled laborer	1.5 (0.90–2.4)	
**Adverse childhood experiences**		
Absent caregiver^b^	0.97 (0.6–1.6)	
Out-of-home experience^c^	1.9 (1.2–3.1)	
Any abuse or neglect^d^	1.7 (1.1–2.6)	
**Young adulthood (18–25 years) experiences**		
Difficulty paying bills or received government support	2.7 (1.6–4.5)	2.9 (1.6–5.5)
Lacked spouse/partner	1.5 (0.97–2.4)	2.0 (1.2–3.5)
Mental health problem^e^	1.9 (1.1–3.3)	2.0 (1.0–3.9)
**Middle adulthood (26–49 years) experiences**		
Underemployed^f^	2.9 (1.8–4.6)	3.0 (1.8–5.1)
Traumatic brain injury	2.8 (1.5–5.4)	2.5 (1.2–5.2)
**Cumulative experiences over three life periods**		
Imprisonment	1.7 (1.3–1.7)	1.4 (1.1–2.0)
Chronic medical condition^g^	1.9 (1.3–2.7)	
Alcohol use problem^h^	1.7 (1.4–2.3)	1.9 (1.3–2.7)
Drug use problem^i^	1.4 (1.2–1.7)	

^a^Final multivariable model developed using a structured modeling approach which evaluated alternative ways of expressing the association between each life course experience and experiencing a first episode of adult homelessness before age 50.

^b^Absent caregiver defined as either a parent or other caregiver who died or a parent who was incarcerated for 12 months or longer.

^c^Out-of-home experience in childhood included placement in the child welfare system, incarceration in the juvenile justice system, homelessness experienced with one’s family, and homelessness experienced after running away or being forced to leave home.

^d^Any abuse or neglect in childhood defined as experiencing neglect, emotional abuse, physical abuse, or sexual abuse.

^e^Mental health problem defined as first reporting any of the following experiences during a given life course period: experiencing serious anxiety, depression, difficulty controlling violent behavior, or hallucinations that were not the result of substance use; attempting suicide; or being prescribed medication by a doctor for psychiatric problems.

^f^Underemployment during a given life course period defined as working at least 20 hours per week less than half the time during that time period.

^g^Chronic medical condition defined as receiving a new diagnosis of any of the following during a given life course period: hypertension; coronary artery disease, myocardial infarction, or angina; congestive heart failure; stroke or transient ischemic attack; diabetes; asthma or chronic obstructive pulmonary disease; arthritis; HIV or AIDS; or cancer.

^h^Alcohol use problem defined as a self-reported history of drinking to get drunk 3 or more times a week during a given life course period.

^i^Drug use problem defined as a self-reported history of using drugs 3 or more times a week.

Multivariable results: After multivariable adjustment, we found an association between several life course experiences that occurred in young and middle adulthood and onset of first homelessness before age 50. Receiving government support or having difficulty paying bills during young adulthood was associated with a higher odds of first homelessness before age 50 (adjusted odds ratio [AOR] 2.9 [95% CI 1.6–5.5]), as was lacking a spouse or partner (AOR, 2.0 [95% CI, 1.2–3.5) and having a mental health problem (AOR, 2.0 [95% CI, 1.0–3.9]) (Table 2). Underemployment during middle adulthood was associated with a higher odds of first homelessness before age 50 (AOR, 3.0 [95% CI, 1.8–5.1]), as was traumatic brain injury (AOR, 2.5 [95% CI, 1.2–5.2]).

For several experiences, the odds of homelessness before age 50 increased as a function of the number of life course periods during which one experienced the event. Each additional life course period of imprisonment was associated with a higher odds of first homelessness before age 50 (AOR, 1.4 [95% CI, 1.1–2.0]), as was each additional period of regular alcohol use (AOR, 1.9 [95% CI, 1.3–2.7]).

Current vulnerabilities: Several measures of current health and functional status differed by age at first adult homelessness. Individuals whose first homelessness occurred at age 50 or older reported a higher prevalence of fair or poor health, at borderline significance (60.1% versus 50.0%, P = .06). This group also reported a higher prevalence of several mental health issues, including experiencing moderate to severe depressive symptoms (44.4% versus 30.5%, P = .008), having a positive screen for PTSD (39.4% versus 23.7%, P = .002), experiencing hallucinations in the past 6 months (19.2% versus 7.9%, P = .003), and being hospitalized for mental health problems in the past 6 months (7.1% versus 2.0%, P = .03). Participants with first homelessness before age 50 had a higher prevalence of at-risk drug use in the past 6 months (57.1% versus 42.1%, P = .006). In terms of functional impairment, IADL impairment was more common among individuals with first homelessness before age 50 (58.6% versus 37.5%, P <.0001); ADL impairment was also more common in this group, though at borderline significance (42.9% versus 33.6%, P = .07). There were no measures of vulnerability that were higher in those with late onset homelessness (Table 3).

**Table 3:** Current vulnerabilities of 350 older homeless adults by age at first homelessness.

Characteristic	First homelessness before age 50 (n = 198)	First homelessness age 50 or older (n = 152)	P value
**Incarcerated in past 6 months, no. (%)**	22 (11.1)	15 (9.9)	0.71
**Health status**			
Fair or poor health^a^, no. (%)	119 (60.1)	76 (50.0)	0.06
Chronic conditions^b^, mean (SD)	1.3 (1.1)	1.4 (1.3)	0.84
**Mental health status and substance use, no (%)**			
Moderate to severe depressive symptoms^c^	87 (44.4)	46 (30.5)	0.008
Post-traumatic stress disorder^d^	78 (39.4)	36 (23.7)	0.002
Hallucinations in past 6 months	38 (19.2)	12 (7.9)	0.003
Trouble controlling violent behavior in past 6 months	18 (9.1)	6 (3.9)	0.06
Attempted suicide in past 6 months	2 (1.0)	0 (0.0)	0.21
Received outpatient mental health treatment in past 6 months	35 (17.7)	21 (13.8)	0.33
Psychiatric hospitalization in past 6 months	14 (7.1)	3 (2.0)	0.03
At-risk drug use^e^	113 (57.1)	64 (42.1)	0.006
At-risk alcohol use^f^	54 (27.4)	36 (23.7)	0.43
**Functional and cognitive status, no (%)**			
ADL impairment^g^^,^^h^	85 (42.9)	51 (33.6)	0.07
IADL impairment^g^^,^^i^	116 (58.6)	57 (37.5)	<0.0001
Mobility impairment^j^	58 (29.3)	36 (23.7)	0.24
Cognitive impairment^k^	49 (24.7)	41 (27.2)	0.61

^a^Health status assessed by self-reported general health using Ware, et al. 1-item health screen

^b^Chronic conditions assessed by self-report of having received a diagnosis from a physician of the following: hypertension; coronary artery disease, myocardial infarction, or angina; congestive heart failure; stroke or transient ischemic attack; diabetes; asthma or chronic obstructive pulmonary disease; human immunodeficiency virus or acquired immunodeficiency virus; or cancer.

^c^Moderate to severe depressive symptoms defined as a Center for Epidemiologic Studies Scale score of ≥22 (range, 0–60; higher scores indicate more problems)

^d^Post-traumatic stress disorder symptoms defined as experiencing ≥3 out of 4 symptoms on the Primary Care PTSD Screen

^e^At-risk drug use defined as Alcohol, Smoking, and Substance Involvement Screening Test (ASSIST) score ≥4

^f^At-risk alcohol use defined as Alcohol Use Disorders Identification Test (AUDIT) score

^g^ADL, activities of daily living; IADL, instrumental activities of daily living

^h^ADL impairment defined as self-reported difficulty performing 1 or more ADLs.

^i^IADL impairment defined as self-reported difficulty performing 1 or more IADLs.

^j^Mobility impairment defined as difficulty walking across the room.

^k^Cognitive impairment defined as a Modified Mini-Mental State Examination score below the 7^th^ percentile (i.e., 1.5 standard deviations below the demographically-adjusted cohort mean).

## Discussion

We found that nearly half of older homeless adults in a population-based cohort became homeless for the first time after age 50, and that the life course experiences and current health and functional status of individuals with first homelessness before age 50 differed in key ways from those whose first homelessness occurred later. Those with first homelessness before age 50 had more adverse life experiences and lower attainment of typical milestones in young and middle adulthood; these experiences were associated independently with earlier onset of first homelessness. Individuals with first homelessness before age 50 also had more current vulnerabilities than those with later onset of homelessness, including mental health problems, substance use problems, and functional impairment. Our findings highlight the divergent pathways to homelessness and differing vulnerabilities among older homeless adults, and suggest the need for strategies that heed these differences in order to prevent and end homelessness among older adults.

Our findings are consistent with earlier research suggesting that many older homeless adults become homeless for the first time in later life [14,15], but extend this earlier work by showing how life course experiences differ among older homeless adults with earlier versus later onset of homelessness. Previous research in a small cohort of older homeless adults found that more than half led relatively “conventional” lives in terms of job and housing histories before becoming homeless late in life [14]. In contrast, those who had less conventional lives—marked by multiple bouts of homelessness and tenuous ties to housing—became homeless on average 15 years earlier, and were more likely to have had substance use problems and to have experienced disruptive childhood events [14].

Our findings build upon and extend these findings in a larger, population-based sample. Consistent with our hypothesis, we found that a history of adverse life experiences and lower attainment of milestones in young and middle adulthood were associated with onset of first homelessness before age 50. These findings are consistent with earlier work conducted in younger samples of homeless adults showing that similar characteristics—mental health problems, substance use problems, socioeconomic difficulty, poor social support—are associated with a higher risk for homelessness [13,42–44]. These earlier studies used case-control designs that matched currently or previously homeless persons with indigent housed individuals in the general population [13,43,44]. As in this previous work, the associations we found do not establish causality, but point to the association between these factors and first homelessness before age 50 among those who remain homeless into their 50s. In some cases, the association may reflect bidirectional relationships; mental health and substance use problems may increase the risk for homelessness by limiting an individual’s ability to maintain employment, housing, and relationships, but homelessness may also worsen or precipitate mental health and substance use problems. Similarly, lower attainment of milestones such as marriage and full-time employment may increase the risk for homelessness due to a lack of social support and income, but may also result from a life disrupted by homelessness [42].

Our findings further suggest that experiencing negative life events during key life course periods may be associated with earlier onset of homelessness. Experiencing socioeconomic difficulty in young and middle adulthood was associated with earlier onset of homelessness, as was lacking a spouse or partner in young adulthood. These life course periods are typically important earning years when individuals establish financial stability and may form long-term partnerships, factors which may decrease the risk of homelessness [42]. Individuals who experience socioeconomic difficulty during these key periods may achieve less resume building, thereby decreasing the likelihood of secure employment and increasing the risk of homelessness. For imprisonment and alcohol use problems, in contrast, the association with earlier onset of homelessness increased for each additional life course period when an individual had these problems, independent of timing. Alcohol use may be more “globally disruptive” across life domains, interfering not only with one’s financial stability but also with one’s social functioning and physical and mental health. Imprisonment has been shown to have negative effects on psychological function and physical health [45,46], and may also affect financial stability and ability to obtain secure employment. Perhaps for these reasons, these experiences may be associated with earlier onset of homelessness regardless of when they occur relative to life course development. As above, these findings show the association between these events and earlier onset of homelessness and cannot establish causality. However, these associations point to how interventions might address risk factors for early onset of homelessness. As financial difficulties during critical life periods may increase the risk of homelessness, interventions at the time of difficulty may be necessary to mitigate this risk; for incarceration and alcohol use problems, interventions throughout the life course may be successful in reducing risk.

Our findings also show that key current vulnerabilities—and, by extension, the need for services to address those vulnerabilities—differ by onset of homelessness. Recent mental health and substance use problems were more common among participants with earlier onset of homelessness, as was difficulty performing instrumental activities of daily living. These adversities may impair the efforts of individuals with earlier onset of homelessness to exit homelessness, and have implications in planning services and interventions for the growing older homeless population.

Housing First Permanent Supportive Housing (PSH), defined as subsidized housing with closely linked or on-site supportive services (e.g., medical, psychiatric, case management, vocational, and substance use services) and low barriers to entry, has been deemed the national strategy for ending homelessness among those with chronic homelessness [47,48]. Chronic homelessness is defined as having a disabling condition and either a year or more of continuous homelessness or four or more episodes of homelessness with a combined length of time of at least 12 months over the previous three years [47,49]. PSH has been found to maintain housing in individuals at high risk of remaining homeless [50,51] and to provide a cost-effective alternative to placement in skilled nursing facilities for older homeless adults [52]. Federal efforts to focus on PSH for chronically homeless individuals are credited with reducing chronic homelessness in major metropolitan areas [53]. The Veterans Health Administrations’ adaptation of PSH as its strategy to address chronic homelessness has resulted in dramatic reductions in chronic homelessness among veterans [54].

While almost half of the participants in our study became homeless after age 50, over half (59.9%) of those with late-life homelessness had been continuously homeless for greater than a year. Given the high prevalence of disabling conditions seen in older homeless adults [3–5], many of these individuals would meet Federal criteria for chronic homelessness. Increasing numbers of older adults are likely to be at risk for chronic homelessness in coming years, due to demographic changes including increasing numbers of Americans over age 50, an increased proportion of low-income older adults and adults from ethnic and racial minority groups, and decreased availability of affordable housing [11]. Among Americans aged 50 and older, 30% of renters and 23% of homeowners are severely cost-burdened, spending 50% or more of their combined household income on housing [11], and placing them at risk of homelessness. Older age is a known risk factor for long-term homelessness amongst those with first-time homelessness [55].

While PSH has been highly successful in ending chronic homelessness, less intensive efforts may be effective for those with fewer adversities. We found that nearly half of older homeless adults first became homeless after age 50, and these individuals had fewer adverse experiences and reached more adult milestones than those with earlier homelessness. Moreover, these individuals had a lower prevalence of current vulnerabilities, including mental health and substance use problems and functional impairments. This finding raises the question of whether some individuals who become homeless after age 50 would respond to less intensive interventions than those with earlier homelessness, particularly if their homelessness were addressed early. Identifying those at highest risk of losing housing in late life and working to prevent housing loss or provide early support to exit homelessness may be an effective strategy to prevent progression to chronic homelessness in these adults.

This study has several limitations. Because we collected life course data cross-sectionally, recall bias may differentially affect participants’ reporting of past events. Individuals who became homeless earlier in life may be more likely to recall negative events compared to those who became homeless later. We dichotomized the cohort at age 50, rather than examining factors associated with longer-term versus shorter-term homelessness. We chose this age cut-off given the rapid increase in the population of homeless persons aged 50 and older and the need to develop strategies to prevent and end homelessness in older adults. Another limitation is the possibility of reverse causality. For participants who first became homeless at age 50 or older, all reported life course experiences occurred before first onset of homelessness. However, for individuals who became homeless earlier, some experiences may have occurred at the same time as or after first onset of homelessness. For this reason, we cannot assess causality, but only association between life experiences and onset of homelessness before age 50. Participants who were homeless in early life and remained homeless after age 50 may differ from those who became homeless earlier in life and exited homelessness. However, our findings point to the key differences in the association of life course experiences in older homeless adults with earlier versus later onset of homelessness. Finally, our study is based on data from one city and may not reflect the situation in other localities. Although there is a high prevalence of housing cost burden among adults aged 50 and older throughout the United States [56], housing costs in San Francisco Bay area are among the highest the country[57]. Oakland has a higher proportion of African Americans than the country overall. African Americans have a higher risk of housing loss, due to effects of housing, education and employment discrimination, and limited intergenerational wealth transfer [56,58]. As a result of these local factors, participants in our study may have experienced relatively fewer adverse events than those who experience homelessness in other parts of the country.

As the population of older homeless adults continues to grow, identifying appropriate services and interventions for this group is important, including a focus on preventing housing loss in late life. These services must be tailored to address the diverse life experiences and needs of the older homeless population.

## Supporting Information

S1 File Dataset Contents. This file provides supplemental information about the variables included in the manuscript dataset.
(RTF)

S1 Table Manuscript Dataset. This spreadsheet includes the data for the variables examined in this paper.
(CSV)

## References

[1] Hahn JA, Kushel MB, Bangsberg DR, Riley E, Moss AR. BRIEF REPORT: the aging of the homeless population: fourteen-year trends in San Francisco. J Gen Intern Med. 2006; 21: 775–778. 16808781 10.1111/j.1525-1497.2006.00493.x PMC1924700

[2] Culhane DP, Metraux S, Byrne T, Stino M, Bainbridge J. The Age Structure of Contemporary Homelessness: Evidence and Implications For Public Policy. Analyses of Social Issues and Public Policy. 2013; 13: 228–244.

[3] Gelberg L, Linn LS, Mayer-Oakes SA. Differences in health status between older and younger homeless adults. J Am Geriatr Soc. 1990; 38: 1220–1229. 2147193 10.1111/j.1532-5415.1990.tb01503.x

[4] Brown RT, Kiely DK, Bharel M, Mitchell SL. Geriatric syndromes in older homeless adults. J Gen Intern Med. 2012; 27: 16–22. 10.1007/s11606-011-1848-9 21879368 PMC3250555

[5] Brown RT, Hemati K, Riley ED, Lee CT, Ponath C, Tieu L, et al Geriatric Conditions in a Population-Based Sample of Older Homeless Adults. Gerontologist. 2016 pii: gnw011 10.1093/geront/gnw011 26920935 PMC5881727

[6] Watson DP, George C, Walker C “Falling through the cracks”: health care needs of the older homeless population and their implications In: Kronenfeld JJ, editor. Care for Major Health Problems and Population Health Concerns: Impacts on Patients, Providers and Policy. pp. 187–204.

[7] Cohen CI. Aging and homelessness. Gerontologist. 1999; 39: 5–14. 10028766 10.1093/geront/39.1.5

[8] Dietz TL. Drug and Alcohol Use Among Homeless Older Adults: Predictors of Reported Current and Lifetime Substance Misuse Problems in a National Sample. J Appl Gerontol. 2009; 28: 235–255.

[9] Yamane DP, Oeser SG, Omori J. Health disparities in the Native Hawaiian homeless. Hawaii Med J. 2010; 69: 35–41. PMC3123142 20540000

[10] Gonyea JG, Mills-Dick K, Bachman SS. The complexities of elder homelessness, a shifting political landscape and emerging community responses. J Gerontol Soc Work. 2010; 53: 575–590. 10.1080/01634372.2010.510169 20865621

[11] Baker K, Baldwin P, Donahue K, Flynn A, Herbert C, et al Housing America's Older Adults: Meeting the Needs of an Aging Population. Cambridge, MA: Joint Center for Housing Studies of Harvard University 2014 Available: http://www.jchs.harvard.edu/sites/jchs.harvard.edu/files/jchs-housing_americas_older_adults_2014.pdf.

[12] Crane M. Homeless Truths: Challenging the Myths about Older Homeless People. London, England: Crisis; 1997.

[13] Susser E, Moore R, Link B. Risk factors for homelessness. Epidemiol Rev. 1993; 15: 546–556. 8174670 10.1093/oxfordjournals.epirev.a036133

[14] Shinn M, Gottlieb J, Wett JL, Bahl A, Cohen A, Baron Ellis D. Predictors of homelessness among older adults in New York city: disability, economic, human and social capital and stressful events. J Health Psychol. 2007; 12: 696–708. 17855456 10.1177/1359105307080581

[15] Crane M, Byrne K, Fu R, Lipmann B, Mirabelli F, Rota-Bartelink A, et al The causes of homelessness in later life: findings from a 3-nation study. J Gerontol B Psychol Sci Soc Sci. 2005; 60: S152–159. 15860792 10.1093/geronb/60.3.s152

[16] Sermons MW, Henry M. Demographics of Homelessness Series: The Rising Elderly Population. Washington, D.C.: Homelessness Research Institute 2010 Available: http://www.endhomelessness.org/page/-/files/2698_file_Aging_Report.pdf.

[17] Crane M. The situation of older homeless people. Reviews in Clinical Gerontology. 1996; 6: 389–398.

[18] Cable N. Life course approach in social epidemiology: an overview, application and future implications. J Epidemiol. 2014; 24: 347–352. 25018148 10.2188/jea.JE20140045 PMC4150004

[19] The Implications for Training of Embracing a Life Course Approach to Health. Rickmansworth, England: International Longevity Centre—UK, World Health Organization 2000 Available: http://www.who.int/ageing/publications/lifecourse/alc_lifecourse_training_en.pdf.

[20] Patterson ML, Moniruzzaman A, Somers JM. Setting the stage for chronic health problems: cumulative childhood adversity among homeless adults with mental illness in Vancouver, British Columbia. BMC Public Health. 2014; 14: 350 10.1186/1471-2458-14-350 24726046 PMC3991866

[21] Padgett DK, Smith BT, Henwood BF, Tiderington E. Life course adversity in the lives of formerly homeless persons with serious mental illness: context and meaning. Am J Orthopsychiatry. 2012; 82: 421–430. 10.1111/j.1939-0025.2012.01159.x 22880980 PMC3422756

[22] Weiser SD, Hatcher A, Frongillo EA, Guzman D, Riley ED, Bangsberg DR, et al Food insecurity is associated with greater acute care utilization among HIV-infected homeless and marginally housed individuals in San Francisco. J Gen Intern Med. 2013; 28: 91–98. 10.1007/s11606-012-2176-4 22903407 PMC3539018

[23] Homeless Emergency Assistance and Rapid Transition to Housing Act of 2009. Definition of homelessness. PL 111–22, Sec 1003. 111th Congress ed: U.S. Congress. 2009.

[24] Dunn LB, Jeste DV. Enhancing informed consent for research and treatment. Neuropsychopharmacology. 2001; 24: 595–607. 11331139 10.1016/S0893-133X(00)00218-9

[25] Hollingshead AB, Redlich FC. Social Class and Mental Illness: A Community Study. Am J Public Health. 2007; 97: 1756–1757. 17895405 10.2105/ajph.97.10.1756 PMC1994199

[26] McLellan AT, Kushner H, Metzger D, Peters R, Smith I, Grissom G, et al The Fifth Edition of the Addiction Severity Index. J Subst Abuse Treat. 1992; 9: 199–213. 1334156 10.1016/0740-5472(92)90062-s

[27] Burt M, Aran L, Douglas T, Valente J, Lee E, Iwen B, et al. Homelessness: Programs and the People they Serve: Findings from the National Survey of Homeless Assistance Providers and Clients, Technical Report. Washington, DC: The Urban Institute. 1999. Available: http://www.urban.org/sites/default/files/alfresco/publication-pdfs/310291-Homelessness-Programs-and-the-People-They-Serve-Findings-of-the-National-Survey-of-Homeless-Assistance-Providers-and-Clients.PDF.

[28] Green HD Jr., Tucker JS, Wenzel SL, Golinelli D, Kennedy DP, Ryan GW, et al Association of childhood abuse with homeless women's social networks. Child Abuse Negl. 2012; 36: 21–31. 10.1016/j.chiabu.2011.07.005 22265902 PMC3659414

[29] National Health and Nutrition Examination Survey: questionnaires, datasets, and related documentation. Atlanta, GA: Centers for Disease Control and Prevention 2009.

[30] Hux K, Schneider T, Bennett K. Screening for traumatic brain injury. Brain Inj. 2009; 23: 8–14. 10.1080/02699050802590353 19096967

[31] Ware J Jr., Kosinski M, Keller SD. A 12-Item Short-Form Health Survey: construction of scales and preliminary tests of reliability and validity. Med Care. 1996; 34: 220–233. 8628042 10.1097/00005650-199603000-00003

[32] Bland RC, Newman SC. Mild dementia or cognitive impairment: the Modified Mini-Mental State examination (3MS) as a screen for dementia. Can J Psychiatry. 2001; 46: 506–510. 11526806 10.1177/070674370104600604

[33] Radloff LS. The CES-D Scale: A self-report depression scale for research in the general population. Applied Psychological Measurement. 1997; 1: 385–401.

[34] Prins A, Ouimette P, Kimerling R, Cameron RP, Hugelshofer DS, Shaw-Hegwer J, et al The primary care PTSD screen (PC-PTSD): Development and operating characteristics. Primary Care Psychiatry. 2003; 9: 9–14.

[35] Babor TF, Higgins-Biddle JC, Saunders JB, Monteiro MG. The Alcohol Use Disorders Identification Test: Guidelines for Use in Primary Care. World Health Organization 2001 Available: http://apps.who.int/iris/bitstream/10665/67205/1/WHO_MSD_MSB_01.6a.pdf.

[36] Humeniuk R, Henry-Edwards S, Ali R, Poznyak V, Monteiro M. The Alcohol, Smoking and Substance Involvement Screening Test (ASSIST): Manual for use in primary care. Geneva: World Health Organization 2010 Available: http://www.who.int/substance_abuse/activities/en/Draft_The_ASSIST_Guidelines.pdf.

[37] Katz S. Assessing self-maintenance: activities of daily living, mobility, and instrumental activities of daily living. J Am Geriatr Soc. 1983; 31: 721–727. 6418786 10.1111/j.1532-5415.1983.tb03391.x

[38] Sullivan G, Dumenci L, Burnam A, Koegel P. Validation of the brief instrumental functioning scale in a homeless population. Psychiatr Serv. 2001; 52: 1097–1099. 11474058 10.1176/appi.ps.52.8.1097

[39] Bravo G, Hebert R. Age- and education-specific reference values for the Mini-Mental and modified Mini-Mental State Examinations derived from a non-demented elderly population. Int J Geriatr Psychiatry. 1997; 12: 1008–1018. 9395933 10.1002/(sici)1099-1166(199710)12:10<1008::aid-gps676>3.0.co;2-a

[40] Mishra G, Nitsch D, Black S, De Stavola B, Kuh D, Hardy R. A structured approach to modelling the effects of binary exposure variables over the life course. Int J Epidemiol. 2009; 38: 528–537. 10.1093/ije/dyn229 19028777 PMC2663717

[41] Murray ET, Hardy R, Hughes A, Wills A, Sattar N, Deanfield J, et al Overweight across the life course and adipokines, inflammatory and endothelial markers at age 60–64 years: evidence from the 1946 birth cohort. Int J Obes. 2015; 39: 1010–1018. 10.1038/ijo.2015.19 PMC4433551 25676237

[42] Shelton KH, Taylor PJ, Bonner A, van den Bree M. Risk factors for homelessness: evidence from a population-based study. Psychiatr Serv. 2009; 60: 465–472. 10.1176/appi.ps.60.4.465 19339321

[43] Caton CL, Shrout PE, Eagle PF, Opler LA, Felix A, Dominguez B. Risk factors for homelessness among schizophrenic men: a case-control study. Am J Public Health. 2004; 84: 265–270. 10.2105/ajph.84.2.265 PMC1615013 8296951

[44] Caton CL, Hasin D, Shrout PE, Opler LA, Hirshfield S, Dominguez B, et al Risk factors for homelessness among indigent urban adults with no history of psychotic illness: a case-control study. Am J Public Health. 2000; 90: 258–263. 10667188 10.2105/ajph.90.2.258 PMC1446149

[45] Wilper AP, Woolhandler S, Boyd JW, Lasser KE, McCormick D, Bor DH, et al The health and health care of US prisoners: results of a nationwide survey. Am J Public Health. 2009; 99: 666–672. 10.2105/AJPH.2008.144279 19150898 PMC2661478

[46] Dumont DM, Brockmann B, Dickman S, Alexander N, Rich JD. Public health and the epidemic of incarceration. Annu Rev Public Health. 2012; 33: 325–339. 10.1146/annurev-publhealth-031811-124614 22224880 PMC3329888

[47] Opening Doors: Federal Strategic Plan to Prevent and End Homelessness Update 2013. Washington, D.C.: United States Interagency Council on Homelessness 2014 Available: https://www.usich.gov/resources/uploads/asset_library/USICH_Annual_Update_2013.pdf.

[48] Housing First in Permanent Supportive Housing. Washington, D.C.: U.S. Department of Houisng and Urban Development 2014 Available: https://www.hudexchange.info/resources/documents/Housing-First-Permanent-Supportive-Housing-Brief.pdf.

[49] Homeless Emergency Assistance and Rapid Transition to Housing: Defining "Chronically Homeless". Washington, D.C.: Department of Housing and Urban Development pp. 75791–75805. 2015.

[50] Stergiopoulos V, Gozdzik A, Misir V, Skosireva A, Connelly J, Sarang A, et al Effectiveness of Housing First with Intensive Case Management in an Ethnically Diverse Sample of Homeless Adults with Mental Illness: A Randomized Controlled Trial. PLoS One. 2015; 10: e0130281 10.1371/journal.pone.0130281 26176621 PMC4503775

[51] Stergiopoulos V, Hwang SW, Gozdzik A, Nisenbaum R, Latimer E, Sarang A, et al Effect of scattered-site housing using rent supplements and intensive case management on housing stability among homeless adults with mental illness: a randomized trial. JAMA. 2015; 313: 905–915. 10.1001/jama.2015.1163 25734732

[52] Bamberger JD, Dobbins SK. A Research Note: Long-Term Cost Effectiveness of Placing Homeless Seniors in Permanent Supportive Housing. Cityscape. 2015; 17: 269–277.

[53] Byrne T, Fargo J, Montgomery AE, Munley E, Culhane DP. The Relationship between Community Investment in Permanent Supportive Housing and Chronic Homelessness. Social Service Review. 2014; 88: 234–263.

[54] Rosenheck R, Kasprow W, Frisman L, Liu-Mares W. Cost-effectiveness of supported housing for homeless persons with mental illness. Arch Gen Psychiatry. 2003; 60: 940–951. 12963676 10.1001/archpsyc.60.9.940

[55] Caton CL, Dominguez B, Schanzer B, Hasin DS, Shrout PE, Felix A, et al Risk factors for long-term homelessness: findings from a longitudinal study of first-time homeless single adults. Am J Public Health. 2005; 95: 1753–1759. 16131638 10.2105/AJPH.2005.063321 PMC1449432

[56] The State of the Nation's Housing. Boston, MA: Joint Center for Housing Studies of Harvard University 2015 Available: http://www.jchs.harvard.edu/sites/jchs.harvard.edu/files/jchs-sonhr-2015-full.pdf.

[57] February 2015 Rent Report. San Francisco, CA: Zumper. 2015. Available: https://www.zumper.com/blog/2015/03/zumper-us-rent-report-february-2015/

[58] Rockeymoore M, Guzman E. The Racial Wealth Gap: African Americans. Washington, D.C.: Center for Global Policy Solutions 2014.

