# Supplementary material for: Pathways to Homelessness among Older Homeless Adults: Results from the HOPE HOME Study
Source: PLoS One. 2016 May 10;11(5):e0155065. doi: 10.1371/journal.pone.0155065 (PMC4862628; doi:10.1371/journal.pone.0155065)
Supplement: S1 File — This file provides supplemental information about the variables included in the manuscript dataset. (RTF) [file pone.0155065.s001.rtf]

Data Set Name	FLASH.LIFECOURSEDATA_PLOS	Observations	350	
Member Type	DATA	Variables	46	
Engine	V9	Indexes	0	
Created	01/26/2016 18:25:34	Observation Length	368	
Last Modified	01/26/2016 18:25:34	Deleted Observations	0	
Protection		Compressed	NO	
Data Set Type		Sorted	NO	
Label				
Data Representation	WINDOWS_64			
Encoding	wlatin1  Western (Windows)			


Engine/Host Dependent Information	
Data Set Page Size	65536	
Number of Data Set Pages	3	
First Data Page	1	
Max Obs per Page	177	
Obs in First Data Page	159	
Number of Data Set Repairs	0	
ExtendObsCounter	YES	
Filename	G:\life course\lifecoursedata_plos.sas7bdat	
Release Created	9.0401M0	
Host Created	X64_7PRO	


Alphabetic List of Variables and Attributes	
#	Variable	Type	Len	Informat	Label	
9	A1	Num	8	6.	A1. Left without food/shelter as child	
10	A2a	Num	8	6.	A2a. Verbal abuse as child	
11	A3a	Num	8	6.	A3a. Physical abuse as child	
12	A4a	Num	8	6.	A4a. Sexual abuse as child	
39	ACC_Chronic	Num	8			
5	B6	Num	8	6.	B6. Homeless as child	
7	B8	Num	8	6.	B8. Were parent(s) incarcerated when you were child	
8	B9	Num	8	6.	B9. Did parent/caretaker die when child	
3	B4a	Num	8	6.	B4a. Foster care	
6	B7a	Num	8	6.	B7a. Ever run away/pushed out of home	
37	Chronic18	Num	8		Any of 8 chronic dz, age 18-25	
38	Chronic26	Num	8		Any of 8 chronic dz, age 26-49	
36	ChronicMinor	Num	8		Any of 8 chronic dz, age<18	
45	HSorGED	Num	8		Education: HS grad, GED or more	
28	LowAttainment18	Num	8		Low soc attainment 18-25: govt assistance or difficulty paying bills	
29	LowAttainment26	Num	8		Low soc attainment 26-49: govt assistance or difficulty paying bills	
35	LowOccupation	Num	8		Low occupational status	
22	MentalDx18	Num	8			
23	MentalDx26	Num	8			
34	MentalProbChild	Num	8			
24	NeglectAbuseChild	Num	8		Any neglect/abuse as child (<18)	
13	Prison18	Num	8		Prison 18-25 (first or most recent)	
14	Prison26	Num	8		Prison 26-50 (first or most recent)	
44	Race_AfroAmerican	Num	8			
16	Regdrink_18	Num	8			
17	Regdrink_26	Num	8			
15	Regdrink_minor	Num	8			
2	Sex	Num	8		Sex: 1=male, 2=female	
46	SingleParent	Num	8		Raised by single parent	
41	SpousePartner18	Num	8		Any Partner age 18-25	
42	SpousePartner26	Num	8		Any Partner age 26-49	
26	TBILOC_18	Num	8		TBI with LOC, age 18-25	
27	TBILOC_26	Num	8		TBI with LOC, age 26-49	
25	TBILOC_MINOR	Num	8		TBI with LOC, age<18	
30	UnderEmployed18	Num	8		worked 20+hrs/wk less than 50% of time, age 26-49	
31	UnderEmployed26	Num	8		worked 20+hrs/wk less than 50% of time, age 26-49	
18	acc_drink	Num	8		sum of regdrink_minor regdrink_18 regdrink_26	
33	acc_drug	Num	8		sum of regdrug_minor regdrug_18 regdrug_26	
32	acc_incarc	Num	8		sum of incarceratedminor incarcerated18 incarcerated26	
4	incarceratedminor	Num	8	6.	B5a. Juvenile justice incarcerated	
20	regdrug_18	Num	8		Regular drug use age 18-25	
21	regdrug_26	Num	8		Regular drug use age 26-49	
19	regdrug_minor	Num	8		Regular drug use as minor	
43	stablehoused1yr	Num	8		>=1 yr since last stable housing	
40	yearshmls	Num	8		Total years homeless	
